# Increased Force Variability Is Associated with Altered Modulation of the Motorneuron Pool Activity in Autism Spectrum Disorder (ASD)

**DOI:** 10.3390/ijms18040698

**Published:** 2017-03-25

**Authors:** Zheng Wang, MinHyuk Kwon, Suman Mohanty, Lauren M. Schmitt, Stormi P. White, Evangelos A. Christou, Matthew W. Mosconi

**Affiliations:** 1 Schiefelbusch Institute for Life Span Studies, University of Kansas, 1000 Sunnyside Ave., Lawrence, KS 66045, USA; zhengwang@ku.edu (Z.W.); minhyuk.kwon@marquette.edu (M.K.); lmschmitt@ku.edu (L.M.S.); 2 Clinical Child Psychology Program, University of Kansas, 1000 Sunnyside Ave., Lawrence, KS 66045, USA; 3 Kansas Center for Autism Research and Training (K-CART), University of Kansas Medical School, Overland Park, KS 66213, USA; 4 Center for Autism and Developmental Disabilities, University of Texas Southwestern Medical Center, Dallas, TX 75390, USA; smohanty3@humana.com (S.M.); Stormi.White@UTsouthwestern.edu (S.P.W.); 5 Department of Applied Physiology and Kinesiology, University of Florida, Gainesville, FL 32611, USA; eachristou@hhp.ufl.edu

**Keywords:** autism spectrum disorder (ASD), index finger abduction, force variability, motorneuron pool, first dorsal interosseus (FDI) muscle, decomposition-based electromyography (dEMG)

## Abstract

Force control deficits have been repeatedly documented in autism spectrum disorder (ASD). They are associated with worse social and daily living skill impairments in patients suggesting that developing a more mechanistic understanding of the central and peripheral processes that cause them may help guide the development of treatments that improve multiple outcomes in ASD. The neuromuscular mechanisms underlying force control deficits are not yet understood. Seventeen individuals with ASD and 14 matched healthy controls completed an isometric index finger abduction test at 60% of their maximum voluntary contraction (MVC) during recording of the first dorsal interosseous (FDI) muscle to determine the neuromuscular processes associated with sustained force variability. Central modulation of the motorneuron pool activation of the FDI muscle was evaluated at delta (0–4 Hz), alpha (4–10 Hz), beta (10–35 Hz) and gamma (35–60 Hz) frequency bands. ASD patients showed greater force variability than controls when attempting to maintain a constant force. Relative to controls, patients also showed increased central modulation of the motorneuron pool at beta and gamma bands. For controls, reduced force variability was associated with reduced delta frequency modulation of the motorneuron pool activity of the FDI muscle and increased modulation at beta and gamma bands. In contrast, delta, beta, and gamma frequency oscillations were not associated with force variability in ASD. These findings suggest that alterations of central mechanisms that control motorneuron pool firing may underlie the common and often impairing symptoms of ASD.

## 1. Introduction

Sensorimotor impairments are common in autism spectrum disorder (ASD) [1,2]. Disrupted sensorimotor developments may be among the earliest emerging symptoms of ASD, and they are associated with increased severity of social-communication, cognitive, and daily living impairments [3,4,5,6]. Sensorimotor deficits also represent an important target for determining physiological processes disrupted in ASD. Specifically, sensorimotor processes are supported by central and peripheral nervous system mechanisms that are relatively well-understood. By determining patterns of sensorimotor deficits in ASD, and clarifying their underlying physiology, studies of sensorimotor control in patients may provide new insights into neurobiological processes associated with the disorder.

Multiple types of sensorimotor abnormalities have been identified in individuals with ASD, including reduced eye movement accuracy [7,8], postural instability [9,10], increased gait variability [11,12], and atypical handwriting [13]. Reduced ability to control force output also has been repeatedly documented in studies of ASD. Specifically, studies of grip force control in ASD have suggested reduced strength [6,14,15], increased sustained force variability, and reduced force accuracy [16,17]. Force control is essential for everyday tasks requiring manual dexterity (e.g., writing, feeding, and buttoning clothes), and thus determining the physiological processes associated with these deficits may identify new targets for treatments aimed at increasing daily living skills and functional independence.

Force control involves excitatory cortical commands relayed from primary motor cortex (M1) to spinal motor neurons which innervate skeletal muscle fibers [18]. Motor neurons and muscle fibers collectively form individual motor units which work together with other motor units within a motorneuron pool to generate force and maintain target force production. Force production during voluntary contractions involves increasing the number of motor units recruited and their rate of discharge action potentials [19,20,21]. During increases in force production, slow-twitch motor units are recruited early and followed by a gradual recruitment of fast-twitch motor units (the “size principle”) [22]. During sustained force production, recruited motor units show an increase in their discharge rate over time to support a constant level of force output [19,23].

Motorneuron pool firing is coordinated by descending central commands generated at multiple frequencies to dynamically ensure precise force production [18]. Simultaneous recordings of brain and muscle during slow isometric force production have identified a low frequency 0–4 Hz delta oscillation “common drive” generated neocortically that modulates motorneuron pool activation [20,24]. The central origin of this drive is not yet determined, though delta oscillations are seen in premotor and supplementary motor areas during force production [25]. Alpha rhythms from 4 to 10 Hz also are observed during healthy individuals’ slow phasic arm movements and isometric force production [26,27,28].

Beta band (10–35 Hz) frequency modulation of the motorneuron pool coincides with a default mode of cortical innervation from contralateral M1 during tasks involving low to moderate force contractions [29,30,31,32,33]. Increased beta modulation of the motorneuron pool is associated with greater force accuracy in healthy individuals suggesting that strengthened M1 cortical communication to skeletal muscles supports motor precision [34,35]. Gamma frequency (35–60 Hz) modulation of the motorneuron pool activity has been documented during individuals’ maximal voluntary force production with its origins likely located in basal ganglia and frontal cortex [30]. Consistent with this hypothesis, studies have reported reduced gamma modulation in untreated individuals with Parkinson’s disease along with a gradual recovery of gamma synchronization after patients received dopaminergic treatment [30,31,36]. Increased gamma power also is associated with reduced variability of sustained force production [31,37,38].

Defining alterations in the central modulation of the motorneuron pool activity associated with the increased force variability previously documented in ASD [16,17,39,40,41] may offer insights into both the musculophysiological processes that underpin motor deficits, and key central and peripheral processes that are disrupted. To address this critical issue, we applied Delsys decomposition-based quantitative electromyography (dEMG; Delsys, Inc., Boston, MA, USA) recording during participants’ isometric index finger abduction test. The Delsys system collects four-channel surface EMG (sEMG) signal from participants’ hand, from which specific skeletal muscle generates force. sEMG time series were then decomposed offline into distinct motor unit action potential trains using Delsys’ decomposition algorithms (v42) [42,43] to allow us evaluating motorneuron pool activity of the muscle, thus further reveal mechanisms of the central modulation of skeletal muscles at the periphery.

**Figure 1:**
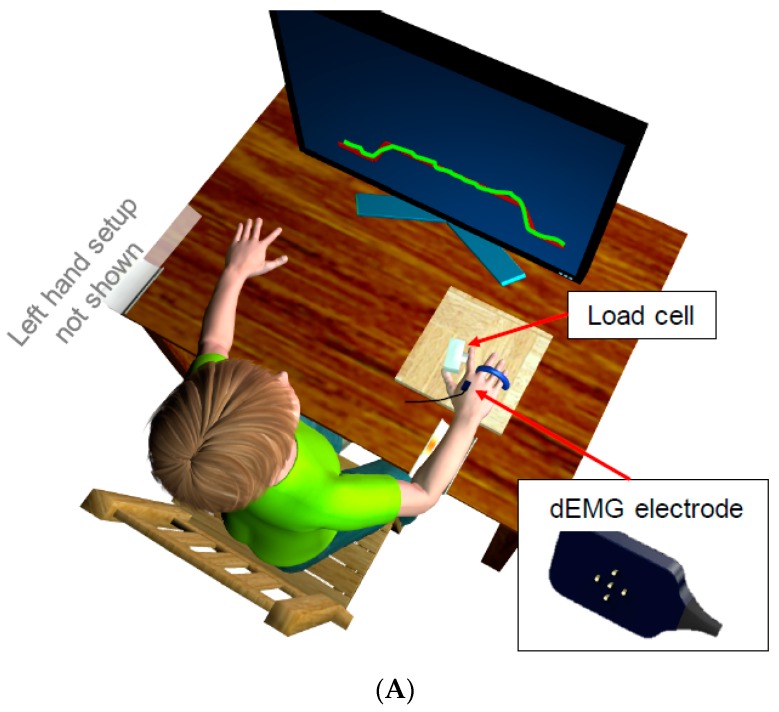

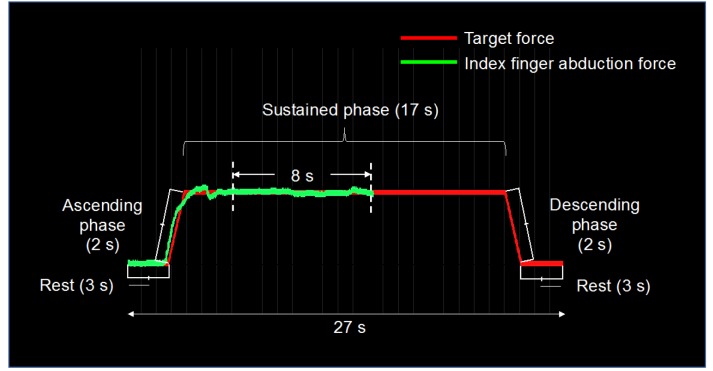
A participant pressed against a load cell with an abductive movement of his right index finger while viewing and tracing the red trapezoidal target template displayed on the monitor (**A**); the participant’s middle, ring and little fingers were isolated and restricted to move on the hand plate. Delsys surface EMG (sEMG) electrode was attached to the back of the participant’s hand in alignment with his first dorsal interosseus (FDI) muscle fibers to record muscle activity during each trial. The left index finger was used for left-handed individuals during the test (setup not shown). The duration of each trial was 27 s (**B**). The red trapezoidal target template includes a 2-s ascending phase, a 17-s sustained phase, and a 2-s descending phase of force production as well as two 3-s rest periods before and after each trial. Participant’s index finger abduction force was displayed on the monitor as a light green line proceeding with time from left to right with its upward displacement representing force increase and downward movement representing force reduction. For each trial, time series of an 8-s force and its corresponding sEMG time series within the sustained phase of force production were randomly selected for evaluation of participants’ behavioral performance (i.e., mean force and standard deviation of force) and motorneuron pool activity of the FDI muscle (i.e., mean discharge rate, discharge rate variability and normalized power of the motorneuron pool activity).

Three study aims were pursued in the present study. First, whereas deficits in controlling grip force may reflect impairments in coordinating force production across multiple effectors, we tested whether individuals with ASD show deficits controlling sustained force produced by one finger in isolation. Second, we examined motorneuron pool firing properties and central modulation of the motorneuron pool at different frequency bands (delta: 0–4 Hz, alpha: 4–10 Hz, beta: 10–35 Hz, and gamma: 35–60 Hz) during isometric index finger abduction in order to characterize neuromuscular properties underlying increased force variability in ASD (Figure 1). Third, we compared the relationships between force production and motorneuron pool activity in both individuals with ASD and healthy controls to determine whether force output is controlled by separate neuromuscular processes in patients. We hypothesized that patients would show increased force variability when attempting to maintain a constant level of force by abducting their index finger. We also predicted that increased force variability in ASD would be associated with increased motorneuron pool discharge rate, increased motorneuron pool discharge rate variability, and atypical central modulation of the motorneuron pool at multiple frequency bands, especially delta, beta and gamma as these frequencies are highly associated with force production and variability in healthy individuals. Consistent with previous findings, we also expected that healthy controls would show a positive association between force variability and 0–4 Hz modulation of the motorneuron pool, and inverse relationships between force variability and modulation of the motorneuron pool at 10–35 and 35–60 Hz. For individuals with ASD, we predicted that these associations would be attenuated suggesting reduced central organization of motorneuron pool activation. Based on prior studies showing that force control impairments may be associated with core symptoms of ASD [15,16,17,39], we also hypothesized impaired force production and modulation of the motorneuron pool activity in individuals with ASD would be related to the severity of their ASD symptoms, including clinically rated social-communication abnormalities [4,5].

## 2. Results

### 2.1. Isometric Index Finger Abduction Force and Variability

**Figure 2:**
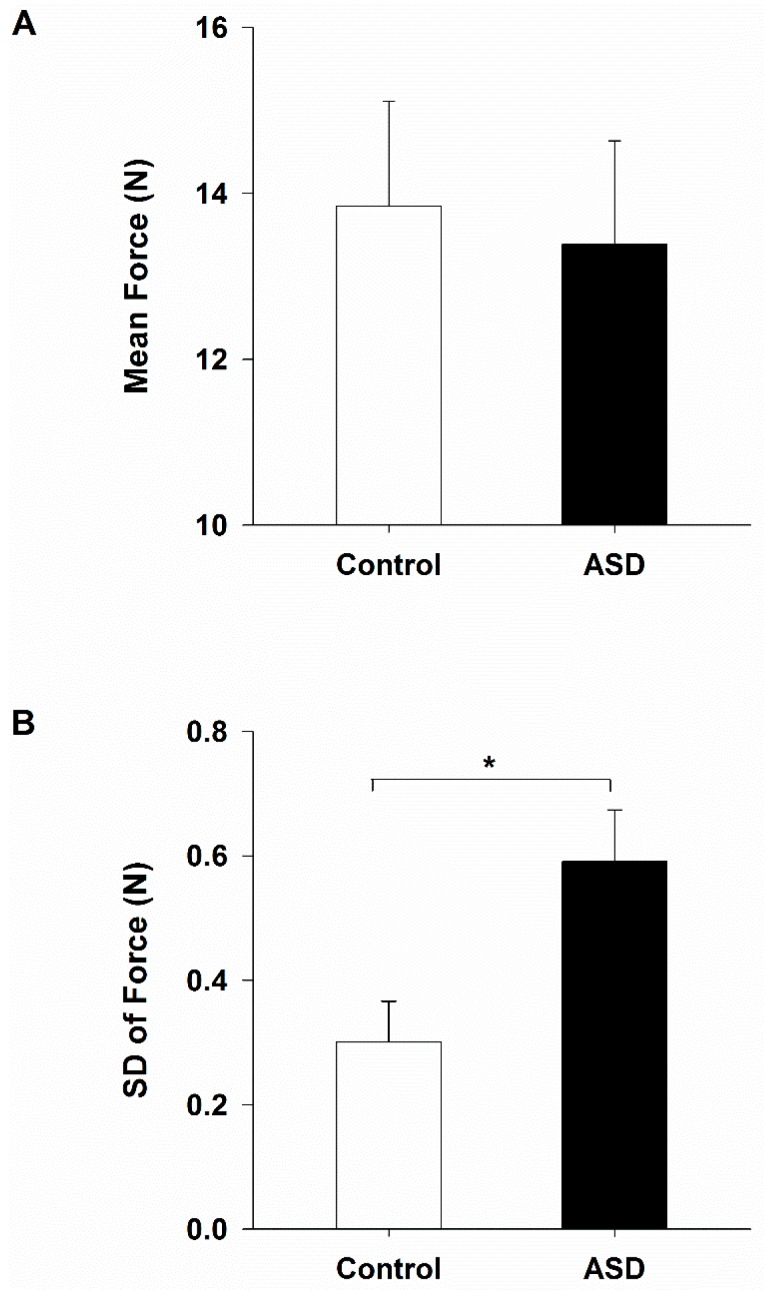
Isometric index finger abduction mean force (**A**); and standard deviation of force (**B**) in healthy controls and individuals with autism spectrum disorder (ASD). Both groups showed similar levels of mean force, although individuals with ASD showed a greater level of sustained force variability than healthy controls. * represents between group significance at the alpha level of 0.05. Error bars represent standard error.

Healthy controls and individuals with ASD showed similar levels of maximum voluntary contraction (MVC) (F_1,30_ = 0.007, *p* = 0.935; CNT = 22.500 N, SE = 2.198 N; ASD = 22.747 N, SE = 2.055 N) and mean sustained force (F_1,30_ = 0.009, *p* = 0.924; Figure 2A) during the test of isometric index finger abduction. Compared to healthy controls, individuals with ASD showed increased sustained force variability (F_1,30_ = 6.641, *p* = 0.015; Figure 2B).

### 2.2. decomposition-Based Electromyography (dEMG) Assessments of Motorneuron pool Activituy of the First Dorsal Interosseous (FDI) Muscle

**Figure 3:**
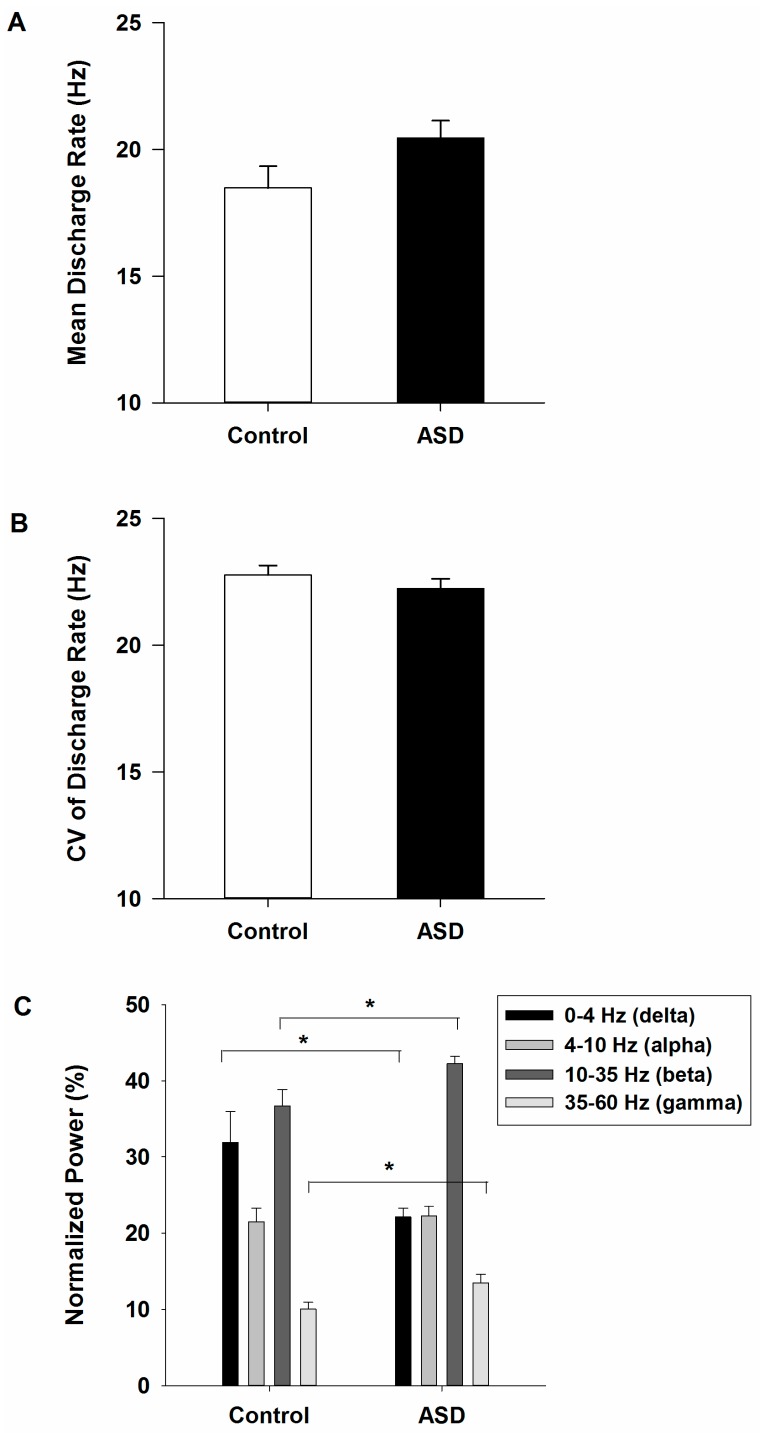
Mean discharge rate (**A**); coefficient of variation of mean discharge rate (**B**); and normalized power (**C**) of the first dorsal interosseous (FDI) muscle motorneuron pool for healthy controls and individuals with ASD. No between group differences were observed for mean discharge rate or discharge rate variability. Individuals with ASD showed greater normalized power at discharge rate of 10–35 (beta) and 35–60 (gamma) Hz, while they also showed lower normalized power at 0–4 (delta) Hz compared to healthy controls. * represents between group significance at the level of 0.05. Error bars represent standard error.

Individuals with ASD and healthy controls showed similar FDI muscle motorneuron pool mean discharge rate (F_1,30_ = 3.316, *p* = 0.079; Figure 3A) and discharge rate variability (F_1,30_ = 1.023, *p* = 0.320; Figure 3B). For normalized power of the motorneuron pool, the main effect of frequency band (F_1.557,45.157_ = 59.379, *p* = 0.000) and the interactive effect of frequency band and group (F_1.557,45.157_ = 5.173, *p* = 0.015) were significant (Figure 3C). Normalized beta power (10–35 Hz) was significantly greater than power at other frequencies, whereas gamma power (35–60 Hz) was significantly lower than other frequency bands (delta (0–4 Hz): 26.976% ± 1.945%; alpha (4–10 Hz): 21.807% ± 1.091%; beta (10–35 Hz): 39.467% ± 1.097%; gamma (35–60 Hz): 11.750% ± 0.737%). Normalized power was significantly lower in individuals with ASD compared to controls at delta band (ASD-Control = −9.787% ± 3.889% , *p* = 0.018), while patients showed greater normalized power than controls at beta (10–35 Hz: ASD-Control = 5.546% ± 2.194%, *p* = 0.017) and gamma (35–60 Hz: ASD-Control = 3.445 ± 1.474%, *p* = 0.027) frequencies. No differences between groups were found for alpha band power.

### 2.3. Relationship between Force Performance and Modulation of the FDI Muscle Motorneuron Pool Activity

**Figure 4:**
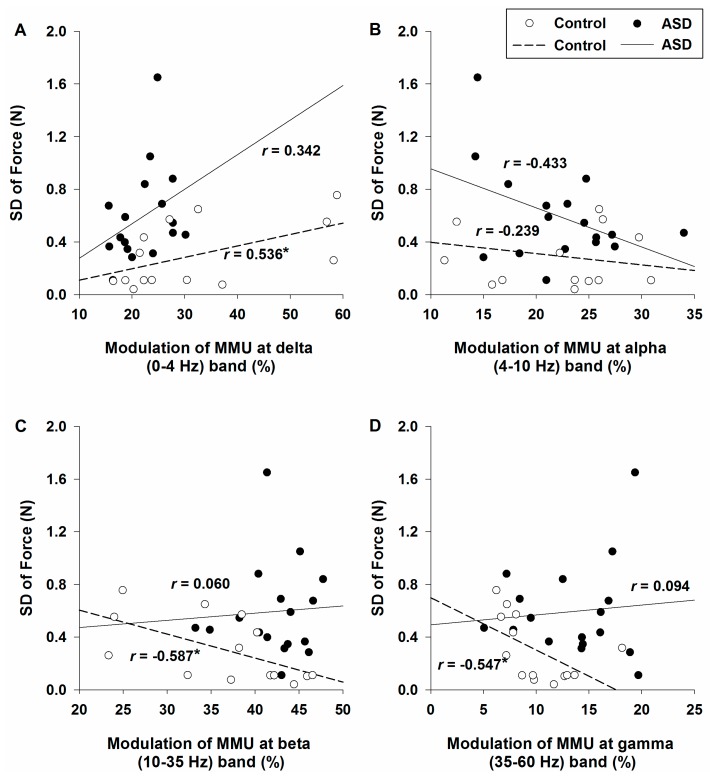
Relationships between the standard deviation of force and motorneuron pool activity of the FDI muscle at frequency bands of: delta, 0–4 Hz (**A**); alpha, 4–10 Hz (**B**); beta, 10–35 Hz (**C**); and gamma, 35–60 Hz (**D**). Behavioral–neuromuscular correlations were observed not significant for individuals with ASD at any frequency bands. Increased force variability was significantly associated with increased modulation of the motorneuron pool firing at 0–4 Hz in healthy controls. Force variability reduction was also significantly associated with increased modulation of motor units at beta (10–35 Hz) and gamma (35–60 Hz) frequency bands in healthy participants. * represents *p* value less than 0.05 and the absolute value of the correlation coefficient (|*r*|) greater than 0.5.

For control participants, increased sustained force variability was associated with increased modulation of the motorneuron pool at delta band (0–4 Hz; Figure 4A) and reduced modulation at frequencies of beta (10–35 Hz; Figure 4C) and gamma (35–60 Hz; Figure 4D) bands. For individuals with ASD, the relationships between force variability and modulation of the motorneuron pool at different frequency bands were not significant. The relationships between sustained force variability and modulation of the motorneuron pool at alpha band (4–10 Hz) were not significant for healthy controls or individuals with ASD (Figure 4B).

### 2.4. Demographic and Clinical Correlations

**Figure 5:**
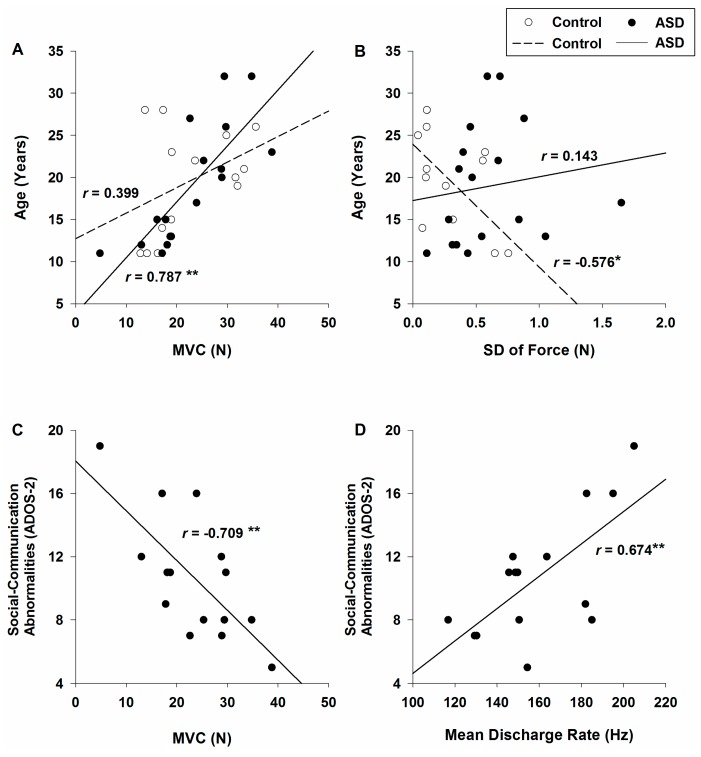
Relationships between age and isometric index finger abduction maximum voluntary contraction (MVC) (**A**) and standard deviation of force (**B**) for both groups. Relationships between clinical ratings of social-communication abnormalities on Autism diagnostic observation schedule-2 (ADOS-)2 and measures of MVC (**C**) and mean discharge rate of motorneuron pool activity (**D**) in individuals with ASD. * represents *p* value less than 0.05, ** represents *p*-value less than 0.01 and the absolute value of the correlation coefficient (|*r*|) greater than 0.5.

Neither force variability nor neuromuscular measurements were associated with intelligence quotient (IQ) scores for healthy controls or individuals with ASD (Table 1). For healthy controls, greater MVC was associated with higher full-scale and performance IQs (Figure 5A). Increased age was associated with reduced force variability for healthy controls (Figure 5B). For individuals with ASD, greater MVCs were associated with increased age but not IQ scores. Higher clinically rated social-communication deficits were associated with lower MVCs (Figure 5C) and greater motorneuron pool mean discharge rate for individuals with ASD (Figure 5D). No correlations were observed between clinically rated restricted, repetitive behaviors ratings and either force or neuromuscular measurements in individuals with ASD.

## 3. Discussion

The present study adds to the growing literature documenting deficits of voluntary hand movements and force production in ASD. Our findings also provide new evidence that motorneuron pool activity power during isometric index finger force production is abnormal in ASD at delta (0–4 Hz), beta (10–35 Hz), and gamma (35–60 Hz) frequency bands. Results further indicate that neuromuscular oscillations at these frequency bands are tightly linked to force control in healthy individuals, but that the central-peripheral communication processes that support the attenuation of force output variability in ASD are distinct and less organized. Taken together, alterations of central modulation of the motorneuron pool activity of the FDI muscle during constant force production may represent a key neurophysiological deficit related to both motor impairment and ASD symptoms.

### 3.1. Altered Force Production in Autism Spectrum Disorder (ASD)

Our findings were consistent with prior studies showing increased variability of manual motor output in ASD during precision gripping [16,17,39], writing [13], object lifting [40,41] and the use of simple finger gestures [44]. As the task of isometric index finger abduction only involves the FDI muscle, our study suggests that failure to precisely adjust force control and motor output in response to visual feedback is evidenced even when actions are restricted to a single muscle group as opposed to requiring coordination across different effectors or muscles. Given that manual motor deficits appear to be associated with increased severity of social-communication symptoms and daily living skills in ASD [3,4,5,6], our findings indicate that the compromised ability of patients to adjust motor output online in response to visual feedback may serve as a component of multiple key clinical issues and functional outcomes.

### 3.2. Altered Motorneuron Pool Activation during Force Control in ASD

Relative to controls, individuals with ASD showed similar levels of FDI muscle motorneuron pool discharge rate (Figure 3A) and discharge rate variability (Figure 3B). While these findings suggest intact central-to-peripheral modulation of skeletal muscles, it remains possible that impairments exist during recruitment of fast-twitch motor units and when increasing the firing rate of low-twitch motor units as has been seen in other developmental disorders [45]. Our findings of increased beta (10–35 Hz) and gamma (35–60 Hz) modulation of the motorneuron pool in individuals with ASD (Figure 3C) indicate atypical central modulation of the FDI muscle during isometric index finger abduction. Increased beta and gamma power may be attributable to increased central noise and/or compensatory modulation processes used to achieve specific motor goals. Increased inherent noise of central oscillators likely would involve elevation of signal power across different frequency bands. As our results showed a reduction of 0–4 Hz delta power in ASD and similar power in ASD and controls at higher frequencies from 4 to 10 Hz, it is likely that increased modulation of the motorneuron pool in ASD reflects compensatory modulation processes used to achieve specific force production.

Motorneuron pool oscillations within the delta band (0–4 Hz) represent a default mode of neural processing with the reductions seen as individuals engage in skilled actions, and associated with greater sustained motor precision [20,24]. At relatively higher force levels, such as the 60% MVC target studied here, output variability increases as delta power is increased due to greater muscle sensitivity to low frequency modulations [21,38,46,47]. In contrast, power of beta (10–35 Hz) and gamma (35–60 Hz) frequency oscillations are increased after visuomotor skill learning and during tasks involving greater attentional and cognitive demands [34,48,49]. Beta oscillations (10–35 Hz) represent co-activation of a large scale central network involving primary sensorimotor and inferior posterior parietal cortex, particularly during visuomotor tests requiring low and medium levels of force production [50,51]. Gamma (35–60 Hz) frequency oscillations, on the other hand, channel primary motor cortex as well as basal ganglia innervation during high level force production and slow phasic movements [26,27,28,30,31,36]. The relationship between reduced force variability and greater modulation of the motorneuron pool at beta and gamma bands thus reflects direct cortical communication to skeletal muscles of the hand that facilitates more precise motor output.

The nature of these compensatory processes remains unclear. Consistent with prior studies, we found that increased modulation of the motorneuron pool at beta (10–35 Hz) and gamma (35–60 Hz) as well as decreased modulation at delta (0–4 Hz) were highly associated with force variability reduction in healthy individuals [21,38,46,47]. These associations were not evident for individuals with ASD, suggesting the neurophysiological processes involved in central modulation of the motorneuron pool activity and control of force output are distinct from healthy individuals. These processes appear sufficient to allow patients to produce a similar level of MVC and mean force as healthy individuals, though they are not sufficient to stabilize motor output during continuous activity. Findings that individuals with ASD utilize unique neurophysiological processes during basic sensorimotor tasks are consistent with prior studies showing that prefrontal-striatal brain systems are more involved in basic movements in ASD than for controls, whereas cerebellar-cortical brain systems typically dedicated to controlling simple visuomotor actions are less involved in movement control in ASD [52,53]. Direct measurements of how modulation of the motorneuron pool varies across different force levels in individuals with ASD may be informative for determining central-to-peripheral mechanisms of force control and hand dexterity deficits in patients.

Unlike with controls, we did not find any relationship in ASD between modulation of motorneuron pool at beta and gamma bands and sustained force variability suggesting that excessive central oscillations were needed to support patients maintaining the target force level, but that these central processes had no effect on the precision of motor output. These findings suggest that central modulations may not be organized in ASD in the same manner as they are in health. For example, it is possible that, for individuals with ASD, fast-twitch and fatigable motor units are not recruited [45] or are recruited earlier than slow-twitch, fatigue resistant motor units. Alternatively, changes in the precise relation between firing rate and the mechanical twitch properties of motor units may also impair force production. In particular, when motor unit firing rates drop to the point where partial fusion of muscle twitches is reduced, muscle contractions become less efficient and more effort must be expended to achieve a force goal. Such increased effort may allow a target force to be reached, but it also likely leads to an increase in the variability of the force output [54].

### 3.3. Neuromotor Deficits, Demographic Characteristics and Clinical Symptoms in ASD

MVC production was more strongly associated with age in ASD compared to controls suggesting that increases in strength during development likely are delayed in ASD. This finding may help explain inconsistencies of prior studies showing both reductions in manual strength [6,14,15] and relatively intact manual strength [16,39]. It also is possible that we did not find MVC differences whereas grip strength has been shown to be impaired in ASD because gripping involves co-contraction of agonist and antagonist hand muscles, central modulation of which might be disrupted in individuals with ASD. It has been documented that motor units from different motorneuron pools respond to central modulation in a synergetic manner during co-contraction due to the fact that these motor units activate as a group according to the task goal and the advantage of this synergetic modulation is to reduce the computational load of central processing [25,55,56]. As our study showed atypical modulation of the motorneuron pool of a single muscle, it is possible that each individual’s hand muscles are modulated in atypical and non-synergetic ways with the resultant effects being augmented during contraction of multiple muscles or co-contractions involving both agonist and antagonist muscles.

Increased age was associated with greater force variability reduction (Figure 5B) in healthy controls, but not in individuals with ASD (Figure 5A), suggesting persistent deficits in controlling force variability in patients. We also found that MVC reductions (Figure 5C) and greater mean discharge rates (Figure 5D) are associated with more severe clinically-rated social-communication abnormalities in ASD. Previous studies also have shown that social-communication symptom severity is related to different aspects of force control deficits in ASD, including reduced sustained force accuracy [39], lower complexity of force outputs [16], and greater target force overshooting during the ascending phase of force production [17]. Together, these findings suggest that social-communication and motor deficits in ASD may reflect common neurodevelopmental mechanisms [4,5], including central processes involved in modulating sensory-motor output. For example, increased motor variability in ASD has been shown to emerge early in development [44], and be linked to failures to understand the movements of others [57] and develop age-appropriate social and cognitive abilities [4,5,6]. Clarifying the timing and course of atypical modulation of motorneuron pool activity in ASD will be important for developing a more mechanistic understanding of motor, social-communication, and cognitive disturbances in patients and their dysmaturation.

### 3.4. Study Limitation

While the present study documents several novel findings useful for understanding neuromuscular processes underpinning force control deficits in ASD, our results should be considered in the context of multiple limitations. First, our sample spans a relatively broad age range. The small sample size may have contributed to insufficient power for characterizing the developmental trajectory of hand MVC and force variability increases in ASD. Further, the high functioning individuals may show less force variability as opposed to patients with lower IQ scores. Second, some of the participants with ASD in our sample may have shown comorbid conditions that are common in this disorder (e.g., Attention deficit hyperactivity disorder (ADHD) and depression). Systematic studies of contributions of these comorbid conditions to force variability increase as well as atypical central modulation of the motorneuron pool at different frequency bands in ASD are needed. Third, interpretations of our current findings, particularly the central origins of delta, alpha, beta and gamma frequency bands will need to be tested by integrating simultaneous measures of central oscillations using electroencephalogram (EEG) or magnetoencephalography (MEG). Such studies, in combination with EMG recording, will allow us to better understand altered central-to-peripheral mechanisms related to both motor and the defining symptoms of ASD. Lastly, antihypertensive and antidepressant medications have unclear effects on psychomotor functioning, with studies documenting both improvement and decline [58,59,60,61]. However, it is unlikely performance was impacted in participants taking either of these medications given that these medications appear to have minimal effect on basic motor functioning and peripheral processes [62].

## 4. Materials and Methods

### 4.1. Participants

Seventeen individuals with ASD and 14 healthy controls matched on age, gender, IQ and handedness (Table 2) performed an isometric index finger abduction test at 60% of their MVC. Tests of 20% and 40% MVC also were administered, though off-line observation of the surface-based EMG signals showed insufficient signal-to-noise ratio; thus, data analyses were conducted only for the 60% MVC condition. IQ was assessed using the Wechsler Abbreviated Scales of Intelligence [63], and handedness was determined using the Edinburgh questionnaire [64]. Individuals with ASD were recruited through community advertisements and local clinical programs. Diagnoses of ASD were confirmed using the ADOS-2 [65] and based on expert clinical opinion using DSM-5 criteria [66]. When possible, the Autism Diagnostic Inventory-Revised (ADI-R) [67] also was used to establish an ASD diagnosis. As parents of several adults with ASD in our study were not available, the ADI-R could only be conducted on 6/17 patients.

Participants with ASD were excluded if they had a known genetic or metabolic disorder associated with ASD (e.g., Fragile-X syndrome, Rett syndrome, and Tuberous sclerosis) or history of non-febrile seizures. Healthy controls were recruited from the community and were required to have a score of 8 or lower on the Social Communication Questionnaire (SCQ) [68]. Control participants were excluded for current or past history of psychiatric or neurological disorders, family history of ASD in first-, second- or third-degree relatives, or a history in first-degree relatives of a developmental or learning disorder, psychosis, or obsessive compulsive disorder.

No participants were taking medications known to affect sensorimotor control at the time of testing, including antipsychotics, stimulants, or anticonvulsants [62]. Seven individuals with ASD were taking antidepressant medication and two were taking antihypertensive medication at the time of testing. No participant had a history of head injury, birth injury, or seizure disorder. After a complete description of the study, written informed consent was obtained from adult participants, and informed parental consent and written assent were obtained for individuals aged less than 18 years. Study procedures were approved by the Institutional Review Board at Children’s Medical Center Dallas (IRB # 062011-010) on 29 April 2012. Participants who are 18 years of age or older provided written consent and minors provided assent in addition to written consent from their legal guardian.

### 4.2. Apparatus and Procedures

Participants were seated in a darkened room facing a 27-inch Dell (Dell Inc., Dallas, TX, USA) LCD monitor (resolution: 1920 × 1080; refresh rate: 120 Hz) located 60 cm in front of them. They sat with their shoulder abducted at 45°, elbow flexed and forearm resting on a customized arm brace (Figure 1A). The arm brace was clamped to a table to keep participants’ arm position stable throughout the test. Participants’ dominant hand (i.e., the left hand was used for left handed individuals) was pronated and laid flat with digits comfortably extended on a hand plate with their middle, ring and little fingers isolated and restricted from moving. This setup only allows isometric index finger abduction at the metacarpophalangeal joint in the horizontal plane, which is a movement exclusively involving contractions of the FDI muscle [19,36]. Participants used the index finger of their dominant hand to press against a precision load cell (capacity: 100 lbf (≈445 N), Miniature Beam Load Cell, Interface Inc., Berwyn, PA, USA) that was securely attached to the hand plate and connected to a Bagnoli-16 surface EMG (sEMG) System (Delsys, Inc., Boston, MA, USA). Participants’ index finger abduction force recorded from the load cell was sampled at a rate of 20 KHz using the Bagnoli-16 sEMG System (Delsys, Inc., Boston, MA, USA).

Prior to testing, each individual’s index finger MVC was measured for their dominant hand. Participants completed three separate 5-s trials in which they were instructed to press against the load cell with as much force as possible by abducting their index finger. The amount of force they generated was displayed on the monitor as a red bar moving upwards with increased force. Participants rested for 1-min between consecutive trials to minimize the effect of muscle fatigue. The average maximum force across trials was calculated as the estimate of each participant’s index finger MVC [16,17,39].

Prior to sEMG sensor attachment, individuals’ skin over the FDI muscle was shaved and cleansed with rubbing alcohol to remove oil, debris and dead skin cells. A reference electrode was taped over the lateral epicondyle of participants’ humerus. Subsequently, a specialized sEMG sensor (dEMG^TM^, Delsys Inc.) was placed on the back of participants’ hand in alignment with their FDI muscle. The sEMG electrode consists of five non-invasive probes (0.5 mm diameter of each) with four arranged in a square and the fifth probe located in the center of the square at a fixed distance of 3.6 mm from each of the surrounding four probes. Pairwise signal subtraction of these probes results in four differential sEMG channels from the FDI muscle. The sEMG time series was then amplified at 1 K, sampled at 20 KHz, and band-pass filtered at 20–450 Hz. Four-channel sEMG time series were decomposed offline into distinct motor unit action potential trains using Delsys decomposition algorithms (v42) [42,43] to evaluate the motorneuron pool activity of the FDI muscle.

During the test, participants viewed a red trapezoidal template displayed on the monitor with a target plateau set at 60% of their own MVC. Participants were instructed to accurately trace the template by adjusting the amount of force generated by abducting their index finger (Figure 1B). Participants’ finger force was displayed as a light green line on the monitor that moved from left to right over time, upward with increased force, and downward with decreased force. Participants adjusted their finger force to keep the light green line as close as possible to the red trapezoidal template throughout the trial. Each trial was 27 s in duration. The red trapezoidal template consisted of three distinct phases, including: (1) a 2-s ascending phase during which participants gradually increased their force; (2) a 17-s sustained phase during which participants attempted to maintain a constant level of force; and (3) a 2-s descending phase during which participants slowly decreased their force. Two 3-s rest phases in addition to a 1 min break were administered before and after each trial to quantify baseline noise of the sEMG signal. During these rest phases, participants kept their index finger away from the load cell. Participants completed a block of three trials for each target force level, as well as practice trials at 30% MVC in order to confirm that they understood task instructions. The test consisted of 3 trials (×3 force levels) alternated with 1-min rest blocks. Total testing time including the electrode attachment, MVC testing, practice trials and task trials lasted 15–20 min.

### 4.3. Data Processing and Analyses

The trial on which the force trace best followed the trapezoidal template was selected for data analyses [38,69]. For each selected trial, the initial and final 5-s of force data was removed from analyses in order to limit variable effects related to initiating sustained force production and fatigue at the end of trials (Figure 1B). Thus, the middle 8-s of abduction force and its corresponding sEMG time series were analyzed off-line using custom-written Matlab programs and Delsys decomposition algorithms (v42) [42,43], respectively.

#### 4.3.1. Force Data

Each raw force trace was digitally filtered using a 4th order low-pass Butterworth filter at a cutoff frequency of 20 Hz and detrended afterwards. Participants’ behavioral performance was quantified using the mean and standard deviation of their 8-s index finger abduction force at 60% MVC.

#### 4.3.2. sEMG Data and Decomposition Procedures for Motor Units’ Activities

**Figure 6:**
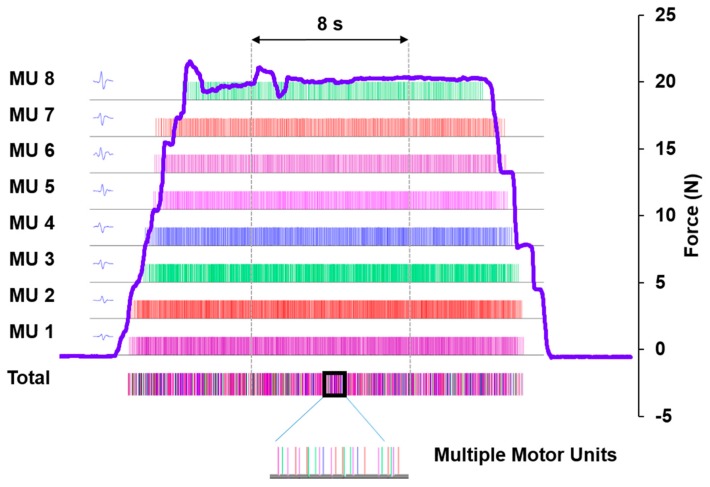
A representative index finger abduction force trace overlaid on eight identified and validated motor units’ action potential firing trains of the FDI muscle. Eight motor units’ action potential patterns were orderly displaced on the left from the smallest waveform located at the bottom to the largest at the top. The total action potential firing train representing motorneuron pool activituy of the FDI muscle was derived by summarizing action potential firing trains of all eight selected motor units. The summarized action potential train thus represents the motorneuron pool activity of the FDI muscle during a trial. The double-sided arrow shows an 8-s period during which force and eight motor unit action potential firing trains were selected for behavioral and FDI muscle activity assessments.

The sEMG time series of each trial was decomposed into distinct motor units. As shown in Figure 6, the action potentials of identified motor units were displayed in order from the smallest to the largest waveforms. To determine the accuracy of the decomposition procedure, a Decompose-Synthesize-Decompose-Compare (DSDC) test was conducted on the action potential train of each motor unit to reduce the incidence of false identification, which further increased the accuracy of validated wave forms [43,70]. Motor units were retained only when their firing was less than 10% of the false identification rate [38,70]. Among those retained motor units, we identified a range of 11–36 and 8–38 motor units for healthy participants and individuals with ASD, respectively.

In order to compare the same number of motor units across groups, the smallest number of validated motor units across participants (*n* = 8) was selected from each trial to represent motorneuron pool activation of the FDI muscle and included in analyses of the mean discharge rate of the motorneuron pool. For those trials with a total number of retained motor units greater than eight, we selected eight motor units based on the following procedures: (1) the smallest and largest motor units were identified as the first (MU1) and the last (MU8) recruited motor units; (2) assigning motor units in the middle of the scale as the fourth (MU4) and fifth (MU5) recruited motor units; (3) assigning motor units in the middle of MU1 and MU4 as the second (MU2) and third (MU3) recruited motor units; and (4) assigning motor units in the middle of MU5 and MU8 as the sixth (MU6) and seventh (MU7) recruited motor units (for detailed procedures, see Appendix A). We then retained eight selected motor units for each trial with the wave forms of their action potentials evenly distributed across the motorneuron pool to ensure an unbiased comparison between individuals with ASD and healthy controls [38]. Motorneuron pool activity of the FDI muscle was derived by summarizing action potential trains of these eight selected motor units.

For each trial, mean discharge rate of the motorneuron pool was examined as the average of the inter-spike intervals. The discharge rate variability of the motorneuron pool was quantified as the coefficient of variation (CV) of the inter-spike intervals calculated using the formula below:

(1) $$\mathrm{CV}\;\mathrm{of}\;\mathrm{Discharge}\;\mathrm{Rate}=\frac{\mathrm{Standar}\;\mathrm{deviation}\;\mathrm{of}\;\mathrm{interspike}\;\mathrm{unterval}}{\mathrm{Mean}\;\mathrm{discharge}\;\mathrm{rate}}\times 100\%$$

Modulation of the motorneuron pool activity was quantified using power spectrum analyses in the frequency domain. Inter-spike intervals of the motorneuron pool were initially transformed into a continuous time series by interpolating each trial [38]. Then, finite Fourier transformation was applied to quantify the power spectrum of the motorneuron pool [38,49,71] with frequencies separated into four bands: delta (0–4 Hz), alpha (4–10 Hz), beta (10–35 Hz) and gamma (35–60 Hz). For each frequency band, the normalized power spectrum of the motorneuron pool was calculated using the formula below:

(2) Normalized Power (%)=(∑power(specific frequency band)Hz)∑power(0−60 Hz)×100%

Each specific frequency bin refers to each of the four frequency bands; thus, normalized power was derived for delta (0–4 Hz), alpha (4–10 Hz), beta (10–35 Hz) and gamma (35–60 Hz) frequency bands.

### 4.4. Clinical Measures

The ADOS-2 was used to confirm participants’ diagnosis and rate ASD symptoms based on the observation of each participant’s behavior [65]. The ADOS-2 is a semi-structured assessment of play, social abilities, communication skills, and imaginative use of materials performed with each individual with ASD by an examiner trained to research reliability. For the ADOS-2, higher scores reflect more severe abnormality in a given domain.

### 4.5. Statistical Analyses

A student *t*-test was used to compare index finger MVCs between individuals with ASD and healthy controls. A series of one-way ANOVAs were conducted to examine between-group (ASD vs. Control) differences on mean sustained force, standard deviation of force, mean discharge rate and discharge rate variability of the FDI muscle motorneuron pool. A 2 (group) × 4 (frequency band: delta (0–4 Hz), alpha (4–10 Hz), beta (10–35 Hz) and gamma (35–60 Hz)) fixed effect repeated measure ANOVA was applied to identify between-group differences in motorneuron pool activations of the FDI muscle across the power spectrum. For all analyses, results were interpreted as significant if *p* < 0.05. Where Mauchly’s test indicated a violation of sphericity, the Greenhouse–Geisser estimate was used to provide a conservative test of ANOVA main and interaction effects.

To determine the relationships between FDI muscle motorneuron pool activity at each frequency band and force variability, Pearson correlations were conducted separately for individuals with ASD and healthy controls. Pearson correlation coefficients also were used to examine the relationships between individuals’ muscle strength (i.e., index finger abduction MVC), force and dEMG measurements found to be different between groups and age, IQ and clinical ratings of ASD severity from the ADOS-2. Results were interpreted as significant if alpha was less than 0.05 and the absolute value of the correlation coefficient (|*r*|) was greater than 0.5.

## 5. Conclusions

Our results demonstrate that individuals with ASD show atypical central modulation of the motorneuron pool of a single hand muscle during isometric index finger abduction. Increased central modulations at beta (10–35 Hz) and gamma (35–60 Hz) frequency bands as well as delta (0–4 Hz) band reduction were all associated with lower force variability in healthy participants, although these relationships were all attenuated in patients. These findings suggest a lack of central communication to skeletal muscles in ASD as well as less organized motor unit recruitment at the periphery. An emerging literature has indicated that early motor developmental abnormalities are among the earliest signs of ASD [3,4,6], combined with our findings that neuromuscular impairments are associated with age and clinically rated social-communication abnormalities in ASD, these results suggest that studies of the development of force control and underlying neuromuscular properties in ASD may provide important insights into neurodevelopmental mechanisms that cause ASD and the emergence of sensorimotor and other core symptoms during childhood.

## Figures and Tables

**Table 1:** Relationships between force and decomposition-based electromyography (dEMG) measurements with demographic, cognitive and clinical dimensions.

**Control (*n* = 14)**	**Age**	**FSIQ**	**PIQ**	**VIQ**		
MVC	0.399	0.590 *	0.640 *	0.477		
SD force	−0.576 *	0.172	0.033	0.270		
Mean discharge rate	0.462	−0.175	−0.067	−0.235		
**ASD (*n* = 17)**	**Age**	**FSIQ**	**PIQ**	**VIQ**	**ADOS.soc.com**	**ADOS.rrb**
MVC	0.787 **	0.296	0.310	0.230	−0.709 **	−0.058
SD force	0.143	−0.300	−0.108	−0.453	0.026	0.329
Mean discharge rate	−0.470	−0.388	−0.332	−0.397	0.674 **	0.1474

MVC: maximum voluntary contraction; SD Force: standard deviation of force; FSIQ: full scale IQ; PIQ: performance IQ; VIQ: verbal IQ; ADOS.sco.com: ADOS-2 social-communication algorithm total; ADOS.rrb: ADOS-2 restricted and repetitive behavior algorithm total; Statistical significance at * α = 0.05; ** α = 0.01 and *r* > |0.5|.

**Table 2:** Demographic characteristics (mean ± SD) of healthy controls and participants with autism spectrum disorder (ASD).

Demographic Characteristics	Control (*n* = 14)	ASD (*n* = 17)	*t*	*p*
Age (yr)	19.57 ± 6.24	18.95 ± 7.14	0.067	0.798
Range	11–28	11–32		
% Male *	85.7 (12/14)	94.14 (16/17)	0.576	0.425
% Right-handed *	92.9 (13/14)	88.23 (15/17)	0.653	0.422
Verbal IQ	112.62 ± 17.74	107.63 ± 17.14	0.589	0.449
Range	82–140	71–126		
Performance IQ	112.69 ± 13.68	106.81 ± 17.68	0.965	0.335
Range	85–133	79–129		
Full-scale IQ	114.77 ± 16.41	108.31 ± 18.34	0.975	0.449
Range	82–138	78–131		

* Chi-square (χ^2^) statistics.

## Abbreviations

ASD	Autism spectrum disorder
ADOS-2	Autism diagnostic observation schedule-2
ADI-R	Autism diagnostic inventory-Revised
sEMG	Surface electromyography
dEMG	Decomposition-based quantitative electromyography
MVC	Maximum voluntary contraction
FDI muscle	First dorsal interosseus muscle
Motor unit	A single motorneuron and the muscle fiber that it innervates
Motorneuron pool	A motorneuron pool consists of all individual motorneurons that innervate a single muscle
Motor unit recruitment or Modulation of motor units	The CNS is responsible for the orderly recruitment of motorneurons through two distinct ways: spatial and temporal recruitment. Spatial recruitment activates more motor units to produce greater force. Temporal recruitment, or rate coding, deals with the frequency or activation rate of motor units firing
Size principle	Henneman’s size principle [22] explains spatial recruitment of motor units, in which motor units are recruited from smallest to largest based on the amount of force production. For smaller force, slow twitch, low-force, fatigue-resistant muscle fibers are activated prior to the recruitment of the fast twitch, high-force, less fatigue-resistant muscle fibers
Motor unit discharge rate	Motor unit discharge rate describes temporal recruitment of motor units represented by spike firing frequency or rate of action potentials

## Appendix A. Motor Unit Selection

MU1 and MU8 always represent the smallest and largest motor units in the motorneuron pool of FDI muscle, respectively. If the total number of recruited MUs is greater than 11, we used formulas below to identify MU2 through MU7 [38]:

$$\mathrm{MU}2=round\left(\frac{1\times \left(a-1\right)}{7}\right)$$

$$\mathrm{MU}3=round\left(\frac{2\times \left(a-1\right)}{7}\right)$$

$$\mathrm{MU}4=round\left(\frac{3\times \left(a-1\right)}{7}\right)$$

$$\mathrm{MU}5=round\left(\frac{4\times \left(a-1\right)}{7}\right)$$

$$\mathrm{MU}6=round\left(\frac{5\times \left(a-1\right)}{7}\right)$$

$$\mathrm{MU}7=round\left(\frac{6\times \left(a-1\right)}{7}\right)$$

where *a* stands for the total number of MUs identified using Delsys decomposition algorithms (v42) [67,68]; and *round* stands for rounding up the element within parentheses to its nearest integer. If the total number of identified MUs is 9, 10 and 11, the above listed formulas cannot be used due to overlapping assignments of specific MUs. For example, it is possible that the smallest MU will be assigned as both MU1 and MU2. If this happens, the following manual selection chart will apply:
